# Platelet factor 4-containing immune complexes induce platelet activation followed by calpain-dependent platelet death

**DOI:** 10.1038/s41420-019-0188-0

**Published:** 2019-06-24

**Authors:** Tatiana A. Nevzorova, Elmira R. Mordakhanova, Amina G. Daminova, Anastasia A. Ponomareva, Izabella A. Andrianova, Giang Le Minh, Lubica Rauova, Rustem I. Litvinov, John W. Weisel

**Affiliations:** 1 Institute of Fundamental Medicine and Biology, Kazan Federal University, 18 Kremlyovskaya St., Kazan, Russian Federation 420008 Russia; 2 Kazan Institute of Biochemistry and Biophysics, FRC Kazan Scientific Center of RAS, 2/31 Lobachevsky str., Kazan, Russian Federation 420111 Russia; 3 Children’s Hospital of Philadelphia, 3401 Civic Center Blvd, Philadelphia, PA 19104 USA; 4 Department of Pediatrics, University of Pennsylvania Perelman School of Medicine, 3401 Civic Center Blvd, Philadelphia, PA 19104 USA; 5 Department of Cell and Developmental Biology, University of Pennsylvania Perelman School of Medicine, 421 Curie Boulevard, Philadelphia, PA 19104 USA

**Keywords:** Mechanisms of disease, Autoimmunity

## Abstract

Heparin-induced thrombocytopenia (HIT) is a complication of heparin therapy sometimes associated with thrombosis. The hallmark of HIT is antibodies to the heparin/platelet factor 4 (PF4) complex that cause thrombocytopenia and thrombosis through platelet activation. Despite the clinical importance, the molecular mechanisms and late consequences of immune platelet activation are not fully understood. Here, we studied immediate and delayed effects of the complexes formed by human PF4 and HIT-like monoclonal mouse anti-human-PF4/heparin IgG antibodies (named KKO) on isolated human platelets in vitro. Direct platelet-activating effect of the KKO/PF4 complexes was corroborated by the overexpression of phosphatidylserine (PS) and P-selectin on the platelet surface. The immune platelet activation was accompanied by a decrease of the mitochondrial transmembrane potential (ΔΨm), concurrent with a significant gradual reduction of the ATP content in platelets, indicating disruption of energy metabolism. A combination of PS expression and mitochondrial depolarization induced by the PF4-containing immune complexes observed in a substantial fraction of platelets was considered as a sign of ongoing platelet death, as opposed to a subpopulation of activated live platelets with PS on the plasma membrane but normal ΔΨm. Both activated and dying platelets treated with KKO/PF4 formed procoagulant extracellular microvesicles bearing PS on their surface. Scanning and transmission electron microscopy revealed dramatic morphological changes of KKO/PF4-treated platelets, including their fragmentation, another indicator of cell death. Most of the effects of KKO/PF4 were prevented by an anti-FcγRII monoclonal antibody IV.3. The adverse functional and structural changes in platelets induced by the KKO/PF4 complexes were associated with strong time-dependent activation of calpain, but only trace cleavage of caspase 3. The results indicate that the pathogenic PF4-containing HIT-like immune complexes induce direct prothrombotic platelet activation via FcγRIIA receptors followed by non-apoptotic calpain-dependent death of platelets, which can be an important mechanism of thrombocytopenia during HIT development.

## Introduction

Heparin-induced thrombocytopenia (HIT) is a common autoimmune disorder that develops in ∼1–5% of patients treated with heparin. Affected individuals generally present with an otherwise unexplained absolute or relative thrombocytopenia, which is a harbinger of arterial or venous thrombosis, occurring in 20–50% of patients^1–3^.

Human platelets are anucleated blood cells that store platelet factor 4 (PF4), a positively charged tetramer belonging to the CXC chemokine family, which is released upon platelet activation. After exposure of patients to heparin, it binds to PF4 and promotes PF4 aggregation, so that they form ultra-large PF4/heparin complexes with antigenic properties. Some patients develop antibodies against PF4/heparin complexes that cause HIT. The reasons for the immune response to the formation of the PF4/heparin complex as an autoantigen remain largely unclear. Anti-PF4/heparin antibodies were detected in 3.1–4.4% of healthy subjects, but occur in ~27–61% of patients after cardiac surgery and in 8–17% of medical and surgical patients treated with heparin. It is estimated that only 5–30% of patients with antibodies against heparin develop HIT^4^.

The pathogenesis of HIT involves circulating PF4/heparin/antibody complexes that bind to the FcγRIIA receptor on platelets and other Fc-receptor-bearing blood cells, such as monocytes and neutrophils^5^. Activation of FcγRIIA causes platelet activation which leads to secretion of the contents of their cytoplasmic granules and to generation of procoagulant microparticles^6,7^. In addition, platelet–neutrophil interactions triggered by HIT antibodies may activate vascular endothelium^8^. PF4/heparin immune complexes also directly activate endothelial cells without involving FcγRIIA, inducing enhanced expression of adhesion molecules such as P- and E-selectins and the release of von Willebrand factor^9^. The combination of direct platelet activation by HIT-related immune complexes through FcγRIIA and transactivation by monocyte and likely endothelial cell-derived thrombin increases expression of phosphatidylserine (PS) and binding of factor Xa to platelets^10^. These consequences lead to generation of thrombin which increases the risk for thrombotic vessel occlusions, such as venous thromboembolism, myocardial infarction, or stroke. Despite the clinical significance, the mechanisms that cause thrombocytopenia in HIT are not well defined^11^.

As the disease progresses, activation of monocytes, endothelial cells, and possibly other cell types contributes to thrombin generation typical for HIT and platelet consumption within thrombi may play a role. However, the extent of thrombocytopenia and severity of thrombosis do not always correlate, which indicates that there are alternative mechanisms for thrombocytopenia other than deposition of platelets within the thrombotic vessels. One of the possible causes of thrombocytopenia in HIT is apoptosis and/or other forms of programmed cell death, which until recently was considered unlikely in a nuclear cells, but has been shown to occur in numerous investigations^12–14^. The study of platelet death induced by pathogenic PF4-containing immune complexes is important to clarify the nature of thrombocytopenia and pathogenesis of thrombotic complications in autoimmune diseases to help reduce the risk and improve outcomes of HIT. Moreover, such studies will make a more general contribution to the understanding of cell death pathways.

The goal of this work was to study the molecular and cellular mechanisms of the interaction of PF4-containing immune complexes similar to those formed in HIT on platelets and their role in activation and death of platelets. We show that PF4-containing pathogenic immune complexes lead to activation of platelets with exposure of phosphatidylserine and expression of P-selectin on the platelet plasma membrane, and mitochondrial depolarization and ATP depletion accompanied by formation of procoagulant phosphatidylserine-expressing microvesicles. Platelet microvesiculation is associated with profound morphological changes and fragmentation of cells. These functional and structural effects of PF4-containing immune complexes on platelets are associated with strong time-dependent activation of protease calpain, but only trace cleavage of caspase 3, suggesting that HIT-like antibodies cause mainly a non-apoptotic calpain-dependent pathway of platelet death that follows the prothrombotic platelet activation.

## Results

### PF4-containing immune complexes induce platelet activation assessed by phosphatidylserine and P-selectin expression

**Fig. 1:**
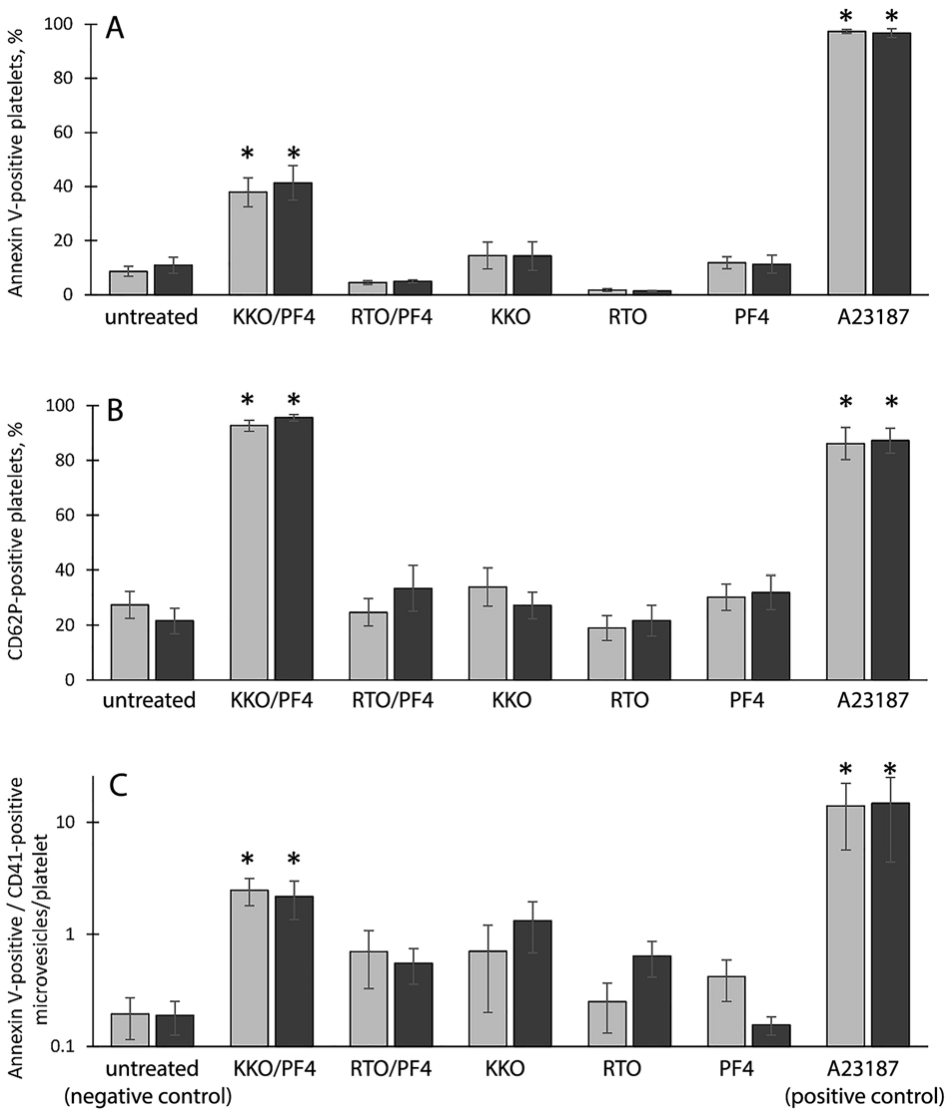
Flow cytometry of platelets and platelet-derived microvesicles. Platelets were incubated for 15 min (light bars) and 60 min (dark bars) under the following conditions: untreated platelets (negative control), platelets treated with the KKO/PF4 and RTO/PF4 complexes, KKO, RTO, and PF4 alone as well as Ca^2+^-ionophore A23187 (positive control). Final concentrations: 10 µg/ml PF4, 50 µg/ml KKO, and RTO, 10 µM Ca^2+^-ionophore. **a** Annexin V-positive platelets as a fraction of all gated platelets taken as 100%: untreated platelets (*n* = 17/13 for 15 min/60 min, respectively), KKO/PF4 (*n* = 16/13), RTO/PF4 (*n* = 3/3), KKO (*n* = 16/13), RTO (*n* = 3/3), PF4 (*n* = 16/13), and A23187 (*n* = 14/12). **b** CD62P-positive platelets as a fraction of all gated platelets taken as 100%: untreated platelets (*n* = 8/7), KKO/PF4 (*n* = 8/7), RTO/PF4 (*n* = 3/3), KKO (n = 8/7), RTO (*n* = 3/3), PF4 (*n* = 8/7), and A23187 (*n* = 8/7). **c** Number of CD41- and Annexin V-double-positive microvesicles normalized by the total number of gated platelets in the corresponding sample: untreated platelets (*n* = 8/9), KKO/PF4 (*n* = 8/9), RTO/PF4 (*n* = 3/4), KKO (*n* = 8/9), RTO (*n* = 3/4), PF4 (*n* = 8/9), and A23187 (*n* = 8/9). *“n”* is the number of experiments with platelets from independent donors. *P < 0.05 compared to a corresponding negative control. The differences between 15 and 60 min are insignificant

Exposure of PS on the platelet surface, a well-known sign of cellular activation and early apoptosis, was measured by the ability of platelets to bind FITC-labeled Annexin V. Flow cytometry of gated platelets revealed that incubation with the immune complexes composed of PF4 and a HIT-pathogenic antibody, KKO, increased the average number of Annexin V-positive platelets ~3–4-fold compared to the untreated control platelets. This effect was revealed after 15 min and maintained up to 60 min incubation (Fig. 1a). The immune complexes composed of PF4 and a nonpathogenic antibody RTO did not change the fraction of Annexin V-positive platelets. No significant activating effect on platelets was observed in the presence of PF4, KKO, and RTO alone, while calcium ionophore A23187 applied as a positive control caused a pronounced PS exposure (Fig. 1a).

P-selectin (CD62P) is a protein transferred to the outer plasma membrane from cytoplasmic secretory α-granules during platelet activation. The KKO/PF4 complexes induced P-selectin exposure in ~95% of platelets after 15 and 60 min of incubation (Fig. 1b); the RTO/PF4 complexes did not induce significant P-selectin expression (~25% and ~34% CD62P-positive platelets after 15 and 60 min of incubation, respectively). The fractions of CD62P-positive platelets in negative controls (untreated platelets as well as platelets treated with PF4, KKO, and RTO alone) were significantly smaller and varied from 19 to 34%, while treatment with calcium ionophore A23187 used as a positive control resulted in formation of ~90% CD62P-positive platelets (Fig. 1b).

### PF4-containing immune complexes induce release of platelet-derived microvesicles

Platelet activation is often followed by production of plasma membrane-derived phospholipid microvesicles 0.1–1 μm in size that bear PS on their surface^15^. Using flow cytometry, we identified microvesicles as small particles <1 μm in size double-stained for a platelet-specific marker CD41 (CD41-positive) (Fig. S2, Q2 gate) and PS (Annexin V-positive). The average number of microvesicles per one platelet went up to ~13-fold after 15 min incubation in the presence of KKO/PF4 compared to control untreated platelets (Fig. 1c). The influence of KKO/PF4 was similar to that of the positive control with A23187. Neither RTO/PF4 immune complexes, nor PF4, KKO, and RTO alone influenced significantly the formation of platelet-derived PS-positive microvesicles.

**Fig. 2:**
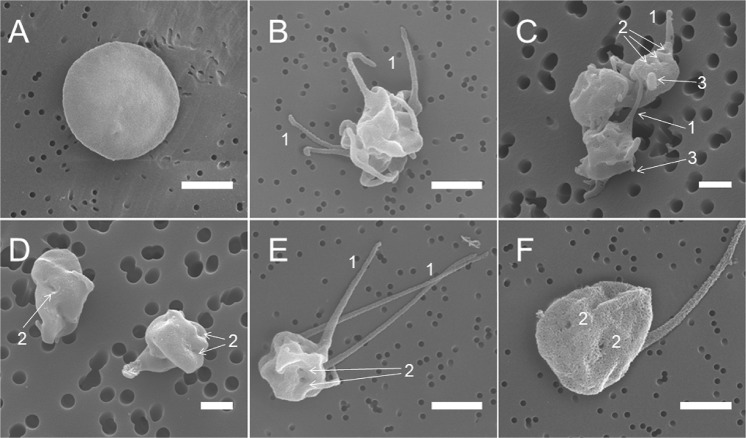
Representative scanning electron micrographs of platelets under various experimental conditions. **a** A control untreated (resting) platelet; **b** a platelet treated with KKO/PF4; **c** an aggregate of platelets treated with KKO alone; **d** platelets treated with PF4 alone; and **e**, **f** a platelet treated with A23187. All the platelet samples were incubated for 15 min at 37 °C. Final concentrations: 10 µg/ml PF4, 50 µg/ml KKO, and 12 µM Ca^2+^-ionophore. Arrows and numbers indicate 1—filopodia/pseudopodia, 2—pores of the open canalicular system, and 3—blebs and knobs. Magnification bars: 1 µm

Formation of microvesicles was also confirmed morphologically using scanning electron microscopy. We observed formation of blebs and knobs on the surface of platelets treated with KKO/PF4 complexes, KKO alone, or PF4 alone which likely represent budding microvesicles (Fig. 2; S3). We noticed grouped or clustered microvesicles ~100–450 nm in diameter in the samples of platelets incubated with KKO/PF4 for 60 min (Fig. S4). These aggregates varied in size from 0.6 to 8.0 µm, with a median of 2.7 µm and a shape varying from elliptical (Fig. S4A, C) to rounded (Fig. S4B, D).

Indirect evidence for platelet vesiculation and perhaps fragmentation was a significant decrease of the absolute number of full-size platelets detected by flow cytometry in the platelet gate. The immune complexes KKO/PF4 decreased platelet populations by about 5-fold after 15 and 60 min incubation, compared to the negative control (Fig. S5). The calcium ionophore A23187 had a similar effect on the platelet counts (about 4- and 5.5-fold after 15 and 60 min incubation, respectively), while the immune complex RTO/PF4 as well as RTO, KKO, and PF4 alone moderately and insignificantly affected the number of full-size platelets in the preparations. These results generally correlate with the number of platelet-derived microvesicles formed under various experimental conditions (Fig. 1c).

### Platelet shape changes induced by PF4-containing immune complexes

Scanning electron micrographs of isolated platelets showed that control untreated platelets had a discoid shape and smooth surface (Fig. 2a; S3A), while platelets treated for 15 and 60 min with the KKO/PF4 complex (Fig. 2b; S3B), KKO alone (Fig. 2c; S3C), PF4 alone (Fig. 2d; S3D), or calcium ionophore A23187 (Fig. 2e,f; S3E, F) demonstrated morphological changes characteristic of platelet activation with a loss of the discoid shape and formation of pseudopodia/filopodia. The pores of the open canalicular systems (OCS) were larger and better visualized in platelets treated with A23187 (Fig. 2f).

**Fig. 3:**
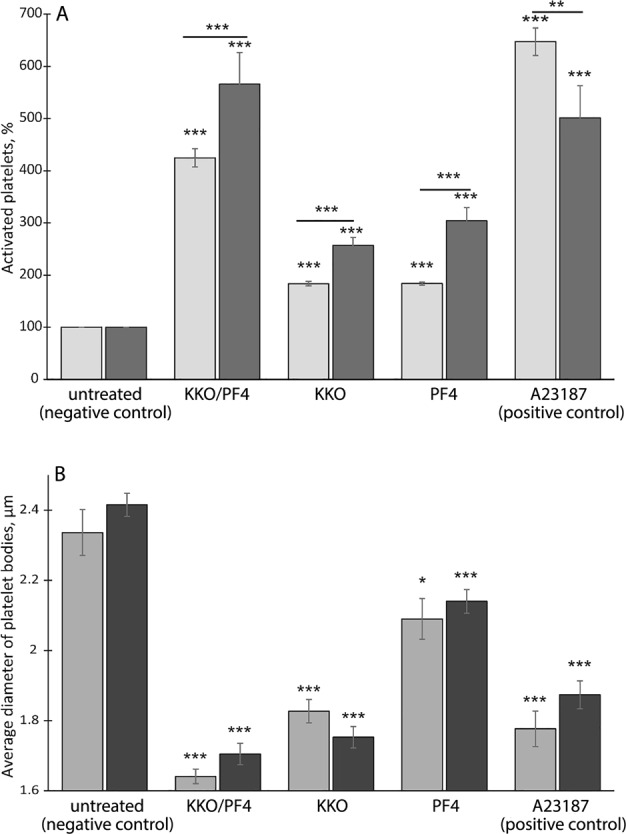
Quantification of platelet activation assessed by scanning electron microscopy. Platelets were incubated for 15 min (light bars) and 60 min (dark bars) under the following conditions: untreated platelets (negative control), treated with KKO/PF4, KKO, PF4, and Ca^2+^-ionophore A23187 (positive control). Final concentrations: 10 µg/ml PF4, 50 µg/ml KKO, and 12 µM Ca^2+^-ionophore. **a** The fraction of activated platelets (with characteristic shape change, see Fig. 2) normalized by the number of activated platelets present in preparations of control untreated platelets (negative control) taken as 100%. **b** Mean diameter of platelet bodies (without membrane protrusions) under various experimental conditions. Number of platelets analyzed varied from 100 to 250 for each experimental condition. Statistical analysis was performed using a chi-square test (**a**) or a two-tailed *t*-test (**b**). Asterisks show statistical significance compared with the corresponding negative control values and between 15 and 60 min incubation (marked by a horizontal line). ***P < 0.001; **P < 0.01; and *P < 0.05 compared to a corresponding negative control

To quantify the incidence of the observed morphological changes, scanning electron micrographs were analyzed to calculate the percentage of platelets with altered morphology (activated platelets) normalized by the number of activated platelets present in the preparations of control untreated platelets taken as 100% (Fig. 3a). The fraction of platelets with the morphological signs of activation was significantly higher in platelets treated with KKO/PF4 complexes, KKO, and PF4 alone or with A23187 compared with control untreated platelets. The fractions of activated platelets in the presence of KKO/PF4 complexes, KKO alone or PF4 alone increased with time, but decreased at 60 min versus 15 min after treatment with A23187 (Fig. 3a) perhaps due to platelet aggregation and/or fragmentation.

As an additional quantitative measure of platelet activation, we determined the size of platelet bodies (without membrane protrusions) under various experimental conditions (Fig. 3b). The average diameter of untreated platelets was 2.34 ± 0.07 and 2.42 ± 0.03 µm after 15 and 60 min incubation, respectively. The average size of platelets treated with KKO/PF4 complex, PF4 alone, KKO alone, or calcium ionophore A23187 decreased significantly compared to the control untreated platelets. Platelets treated with KKO/PF4 had the smallest average size of platelet bodies (1.64 ± 0.02 and 1.71 ± 0.03 µm at 15 and 60 min incubation, respectively). The diameter of platelets treated with PF4 alone was smaller compared to the size of untreated platelets, but larger than the diameter of platelets treated with KKO alone, KKO/PF4, or A23187 (Fig. 3b, Table S1).

### Ultrastructural characterization of platelets induced by PF4-containing immune complexes

**Fig. 4:**
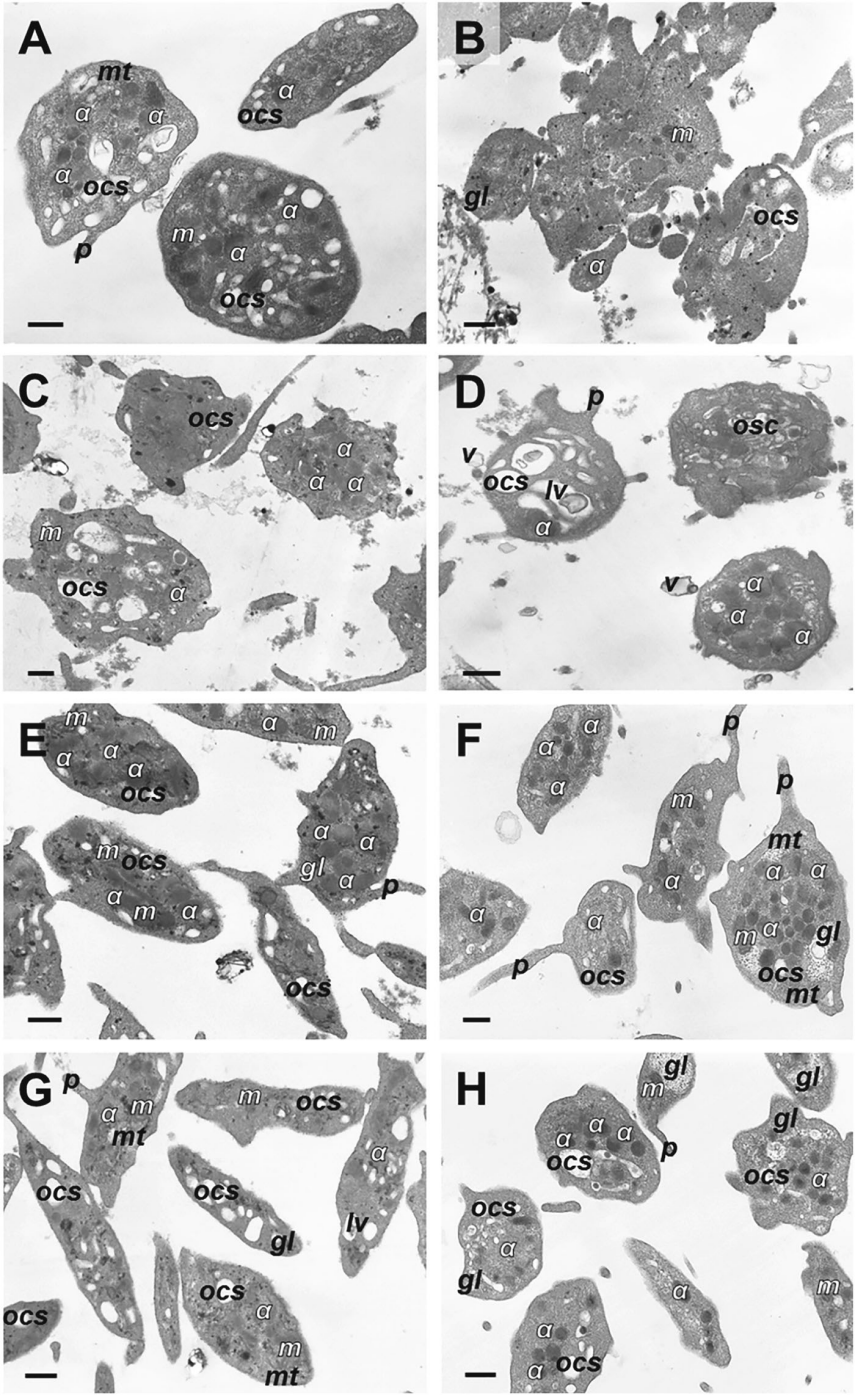
Representative transmission electron micrographs of platelets under various experimental conditions. **a** Control untreated resting platelets, **b** platelets treated with Ca^2+^-ionophore A23187, **c**, **d** platelets treated with KKO/PF4 for 15 min (**c**) and 60 min (**d**), **e**, **f** platelets treated with KKO alone for 15 min (**e**) and 60 min (**f**), **g**, **h** platelets treated with PF4 alone for 15 min (**g**) and 60 min (**h**). Final concentrations: 10 µg/ml PF4, 50 µg/ml KKO, and 12 µM Ca^2+^-ionophore. Designations: *α*, α-granules; *gl*, glycogen granules; *lv*, lytic vacuole; *m*, mitochondria; *mt*, microtubules; *ocs*, open canalicular system; *p*, pseudopodium; *v*, microvesicles. Magnification bars: 0.5 μm. KKO/PF4 complexes (**c**, **d**) induce profound ultrastructural changes in platelets similar to those observed in the positive control (**b**), while platelets treated with KKO and PF4 alone (**e**–**h**) remain largely unperturbed, as in the negative control (**a**)

Transmission electron microscopy of nonactivated resting platelets showed typical morphological characteristics, such as discoid or round shape and a smooth membrane. The linear dimensions were 2–4 μm (Fig. 4a). The peripheral zone contained the dense tubular channel system (microtubules). In the matrix of the cytoplasm electron-dense round α-granules and randomly dispersed or clustered glycogen granules were visualized. Mitochondria with electron-dense matrix and a few cristae were distinctly differentiated from other organelles. In some cells *λ*-granules (lysosomes) in the form of vacuoles were found. We visualized also the OCS connected to the plasma membrane and composed of small vacuoles and lining channels that formed a branched system throughout the platelet body. Platelet stimulation by calcium ionophore A23187 resulted in dramatic morphological changes (Fig. 4b). Platelets acquired an irregular winding shape due to formation of plasma membrane invaginations and protrusions. The lumens of the OCS were enlarged and only a few mitochondria were left. Most α-granules disappeared.

After activation by the KKO/PF4 complex, platelets became structurally heterogeneous (Fig. 4c, d). Some cells were characterized by the centralization of α-granules in the platelets body, fewer mitochondria, and single or clustered glycogen granules randomly dispersed in the cytoplasm. We observed also platelets with slightly higher electron-dense cytoplasm and hypertrophy of the OCS that formed very large lumens and vacuoles, containing various inclusions, such as granules, membrane components, and loose-grained inclusions. There were also some “amoeba-like” platelets with few organelles with diverse size and shape. Platelets incubated with PF4/KKO for 15 or 60 min had similar ultrastructural characteristics.

Incubation of platelets with KKO alone for 15 and 60 min caused minor ultrastructural changes (Fig. 4e, f). The platelets were characterized by few membrane protrusions, enlarged OCS, and formation of some vacuoles that might contain granular inclusions. In some cells, secretory α-granules were grouped near the center of the platelet body and granules of glycogen-formed clusters. The ultrastructural changes induced by PF4 alone for 15 and 60 min were quite minor (Fig. 4g, h) and even lesser or similar to those induced by KKO. Platelets had a wavy membrane, enlarged OCS, and clusters of glycogen granules. Other platelet components were basically unchanged. Platelets in all samples except the control formed pseudopodia of varying lengths.

In the platelet preparations analyzed with transmission electron microscopy, we found monovesicular (Fig. S6A, D, G–I) or multi-vesicular (Fig. S6B, C, E, F) particles either associated (Fig. S6D–G) or not associated (Fig. S6A–C,H, I) with the platelet plasma membrane. The microvesicles formed after incubation of platelets with the immune complex KKO/PF4 varied in size from 0.07 to 0.9 µm (average 0.3 ± 0.02 µm) and were characterized by membrane-surrounded electron-transparent content, occasionally containing granules of glycogen (Fig. S6G) and loose-grained inclusions (Fig. S6H, I).

### PF4-containing immune complexes induce mitochondrial depolarization and ATP depletion in platelets

**Fig. 5:**
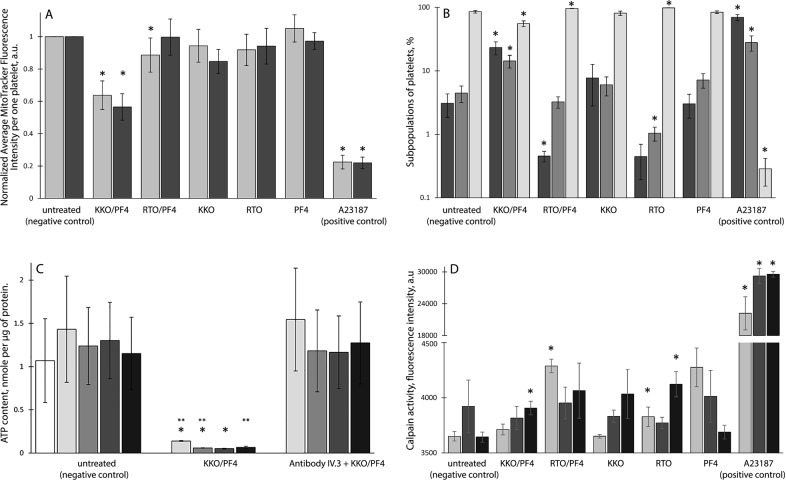
Characterization of platelet functionality under various experimental conditions. **a** Flow cytometry of platelets stained with a ΔΨ_m_-sensitive dye MitoTrackerDeepRed after 15 min (light bars) and 60 min (dark bars) of incubation in untreated platelets (*n* = 15/13 for 15/60 min, respectively) or platelets treated with KKO/PF4 (*n* = 15/13), RTO/PF4 (*n* = 3/3), KKO (*n* = 15/13), RTO (*n* = 3/3), PF4 (*n* = 15/13), and Ca^2+^-ionophore A23187 (*n* = 13/12). **b** Subpopulations of platelets double-stained with a ΔΨ_m_-sensitive dye MitoTrackerDeepRed and FITC-Annexin V normalized by the total number of gated platelets taken as 100%. Platelets were segregated into three groups: MitoTracker-negative/Annexin V-positive or “dead” (dark bars), MitoTracker-positive/Annexin V-positive or “live activated” (gray bars), and MitoTracker-positive/Annexin V-negative or “live resting” (light bars). Platelets were incubated 15 min under the following conditions: untreated platelets (*n* = 15); treated with KKO/PF4 (*n* = 15); RTO/PF4 (*n* = 3); KKO (*n* = 15); RTO (*n* = 3); PF4 (*n* = 15); and Ca^2+^-ionophore A23187 (*n* = 13). **c** ATP content in platelets analyzed after 0 min (white bars), 30 min (light grey), 60 min (gray), 120 min (dark gray), and 180 min (black) of incubation under the following conditions: untreated platelets (negative control), treated with KKO/PF4 without and with pretreatment (5–7 min, 37 °C) of platelets with a monoclonal antibody IV.3 against the FcγRIIA receptor. *P < 0.05 compared to a corresponding time point of the negative control, **P < 0.05 compared to a corresponding time point in the presence of the anti-FcγRII antibody IV.3. **d** Calpain activity in platelets measured in the presence of cell-penetrating fluorogenic calpain substrate after 15 min (light bars), 60 min (dark bars), and 180 min (black bars) of incubation in untreated platelets (negative control) or platelets treated with KKO/PF4, RTO/PF4, KKO, RTO, PF4, and Ca^2+^-ionophore A23187. In **a–d**: Final concentrations: 10 µg/ml PF4, 50 µg/ml KKO and RTO, and 10 µM Ca^2+^-ionophore. “*n”* is the number of experiments with platelets isolated from three independent donors for each experimental condition. *P < 0.05 compared to a corresponding negative control

To reveal alterations of energy metabolism in platelets, we determined changes in the ΔΨ_m_ and intracellular ATP content under various experimental conditions. The fluorescence intensity of platelets stained with a ΔΨ_m_-sensitive dye was significantly suppressed when platelets were treated with the KKO/PF4 complex, as well as with calcium ionophore A23187 used as a positive control (Fig. 5a). The RTO/PF4 immune complex as well as KKO, RTO, or PF4 alone had no visible effect on the ΔΨ_m_ in platelets except a moderate and transitory reduction of ΔΨ_m_ in the presence of RTO/PF4 at 15 min of incubation.

To establish the relationship between the loss of ΔΨ_m_ and platelet functionality, gated platelets were analyzed using flow cytometry after double-staining with FITC-Annexin V (for PS expression) and MitoTracker DeepRed FM (for ΔΨ_m_). Based on the results platelet populations were segregated into three groups: (i) ΔΨ_m_-negative but PS-positive (presumably dead or dying platelets); (ii) ΔΨ_m_-positive and PS-positive (presumably live activated platelets); (iii) ΔΨ_m_-positive but PS-negative (presumably live resting and/or refractory platelets). After 15 min of incubation, the KKO/PF4 complex induced an increase in the subpopulations of “dead” and “activated” platelets with a corresponding reduction of the fraction of “resting” platelets with normal ΔΨ_m_ not expressing PS (Fig. 5b). Incubation of platelets with the KKO/PF4 complex up to 60 min confirmed these results (Fig. S7).

Incubation for 15 and 60 min with the RTO/PF4 complex, unlike KKO/PF4, did not increase the subpopulations of “dead” and “activated” platelets (Fig. 5b; S7). The effects of PF4, KKO, and RTO alone were similar to the negative control with untreated platelets, while calcium ionophore A23187 used as a positive control caused a strong time-independent increase of the fractions of both “dead” and “activated” platelets (Fig. 5b; S7).

The KKO/PF4 complexes reduced dramatically the content of ATP in platelets compared to the corresponding untreated control platelets; the drop was ~10 and 20-fold after 15 and 60 min, respectively, and maintained up to 180 min incubation (Fig. 5c). Remarkably, pretreatment of platelets with the monoclonal antibody IV.3 against the Fcγ receptor lessened significantly the ATP depleting effect of KKO/PF4 complexes on platelets.

### Effects of PF4-containing immune complexes on the activity of caspases 3/7 and calpain in platelets

To investigate if platelets activated with PF4-containing immune complexes undergo an apoptotic death pathway, we measured the activity of the apoptotic effector enzymes caspases 3/7. Flow cytometry revealed no caspase-3/7 activating effect on platelets in the presence of the PF4-containing immune complexes with KKO or RTO as well as with PF4 and KKO alone compared with calcium ionophore A23187, which caused a ~100-fold increase of the average number of caspase 3/7-positive and CD41-positive events compared to the untreated control platelets (32 ± 6% versus 0.32 ± 0.07%, respectively) (Fig. S8). Unlike flow cytometry, the Western blot analysis of platelet lysates revealed a faint band of cleaved caspase 3 in platelets treated with the KKO/PF4 complexes (Fig. S9); this band comprised 15 ± 3% of the total procaspase/caspase content. Such a band was not formed under any other experimental conditions except for the calcium ionophore A23187 (positive control), which induced almost complete conversion of the procaspase 3 (32 kDa) to the active cleaved caspase 3 (17 kDa).

As an alternative to caspases, we measured the activity of a thiol protease calpain in platelets under different experimental conditions. The KKO/PF4 complexes induced a moderate but significant time-dependent calpain activation in platelets that reached a maximum after 180 min of incubation (Fig. 5d), compared with the corresponding negative controls (untreated platelets). Some degree of calpain activation was also observed in platelets treated with RTO/PF4 complexes and RTO alone, but these effects were seen only after 15 min of incubation for RTO/PF4 and 180 min for RTO alone. The effects of KKO and PF4 alone were indistinguishable from the negative control with untreated platelets. As expected, Ca^2+^-ionophore A23187 caused a strong activation of calpain in platelets (Fig. 5d).

## Discussion

Direct activation of platelets by immune complexes containing PF4 is a key pathogenic mechanism of HIT. Platelets activated by the HIT-immune complexes induce formation of small and large thrombi comprising the main life-threatening complication of HIT. The immune platelet activation in HIT is mediated through the interaction of the Fc region of IgG with the FcγRIIA receptor on the platelet surface^10,16–20^. Cross-linking and clustering of FcγRIIA turns on a signaling pathway that ultimately leads to dense granule secretion^21^, followed by the release of PF4 and formation of new HIT immune complexes, thus forming a vicious circle^7,22,23^. Platelet activation is also accompanied by intense generation of thrombin on the negatively charged phospholipids exposed on the outer membrane, comprising another self-activating feedback loop^10,24–28^. Direct immune activation of platelets through FcγRIIA combined with the thrombin-mediated pathway leads to P-selectin expression and formation of procoagulant extracellular microvesicles^7,27,29–31^. As a negative feedback loop of platelet activation induced by HIT-related immune complexes, the FcγRIIA receptor undergoes calpain-dependent proteolytic cleavage and inactivation^7,32,33^. Because calpain has been shown to cleave cytoskeletal proteins and induce platelet death, it is possible that the interaction between HIT-immune complexes and the FcγRIIA receptor contributes to clearance of platelets from the circulation, thus exaggerating thrombocytopenia, in addition to platelet consumption during microthrombosis^7^.

Despite the great pathogenic importance, little is known about structural and functional details of the interactions between HIT immune complexes and platelets and how these interactions directly and indirectly promote thrombosis. Even less is known about delayed effects of the PF4-containing immune complexes on the development of HIT and platelet fate.

Results of the present study have confirmed that platelets undergo strong direct activation by PF4-containing pathogenic HIT-related complexes via the FcγRIIA receptor. This platelet activation is accompanied by a significant increase in the fraction of platelets with exposed phosphatidylserine and P-selectin expression. The biochemical signs of platelet activation induced by the immune complexes composed of PF4 and pathogenic HIT-related antibodies are accompanied by morphological changes that include shrinkage of platelet bodies and formation of membrane protrusions. In addition, flow cytometry combined with scanning and transmission electron microscopy shows that the activated platelets form extracellular procoagulant microvesicles <1 μm in size that express PS. In conformity with platelet vesiculation and partial fragmentation, treatment with the HIT-like immune complexes resulted in a decrease in the number of platelets in the sample, simulating thrombocytopenia.

The main late effects of the HIT-like immune complexes on platelets include a large structural heterogeneity with diverse size and shape, resulting from fragmentation of the platelets. Disintegration of platelets induced by the immune complexes is accompanied by energetic exhaustion, manifesting as mitochondrial depolarization, and a drop in the ATP content, which is similar to the fatal dysfunction and fragmentation of platelets stimulated by thrombin^34^. Fragmentation of activated platelets suggests an underappreciated mechanism for the low platelet counts observed in HIT.

The aggregate of morphological and biochemical changes in platelets revealed after incubation with the HIT-like immune complexes (dramatic shape change, platelet fragmentation and microvesicle formation, phosphatidylserine exposure, the loss of mitochondrial transmembrane potential, and ATP depletion) suggest that platelets undergo some sort of cell death pathway^12,35,36^. We hypothesized that HIT-related immune complexes trigger signaling cascades and induce apoptotic-like events that could be implicated in the pathogenesis of immune thrombocytopenia. To test this hypothesis, we analyzed the activity of executioner caspases 3 and 7 which had been shown to mediate platelet apoptosis via the intrinsic mitochondrial pathway^35–41^. However, we have found that caspases 3 and 7 are either not activated at all (Fig. S8) or very weakly activated (Fig. S9), suggesting that the platelet death pathway induced by HIT-like immune complexes is mostly caspase-independent.

As an alternative for caspases we detected a significant increase of the calpain activity in platelets incubated with HIT-like immune complexes. Calpains are calcium-dependent cysteine proteases that play a key role in platelet activation^42^; importantly, activation of calpain precedes apoptosis^43–45^. In addition, calpain activation has been shown to be concurrent with mitochondrial dysfunction and involved in cell death^45,46^. These earlier findings correlate strongly with our data and they support a notion that platelet activation induced by HIT-like immune complexes turns into a non-apoptotic calpain-dependent cell death pathway^43,45,47^.

In summary, our data show that HIT-like immune complexes interact with platelets via the FcγRIIA receptor and trigger signaling cascades, leading to platelet activation, making platelets highly procoagulant and thrombogenic. This activation later turns into calpain-dependent cell death, likely comprising an additional pathogenic mechanism for removal of activated platelets from the circulation and exaggerating low platelet counts in HIT.

## Materials and methods

### Reagents

Human recombinant PF4 and monoclonal IgG2bk anti-human PF4 HIT-like pathogenic antibodies (KKO), non-pathogenic antibodies (RTO) and anti-human CD32 (FcγRII) antibody, and clone IV.3, were expressed, purified, and characterized as described earlier^48^. Calcium ionophore A23187, Sepharose 2B, and ATP Bioluminescent Assay Kit were from Sigma-Aldrich (USA), hexamethyldisilazane was from Electron Microscopy Sciences (USA); propylene oxide was from Sigma (Germany) and Epon 812 was from Fluka (Switzerland); MitoTracker DeepRed FM, CellEvent™ Caspase-3/7 Green Detection Reagent, and SYTOX^®^ AADvanced™ dead cell stain were from Invitrogen (USA); PE-conjugated monoclonal antibodies to CD41 were from Life Technologies (USA); Annexin V-FITC was from BioLegend (USA); calibration latex beads were from Spherotech (USA); uranyl acetate and lead citrate were from Serva (Germany); Calpain Substrate IV was purchased from Calbiochem (USA).

### Isolation and characterization of platelets

Platelets were freshly isolated from the blood of 20 healthy donors not taking aspirin, nonsteroidal anti-inflammatory drugs or antibiotics, or other medications affecting platelet function at least 2 weeks before the blood withdrawal. The study was approved by the Ethical Committee of Kazan State Medical Academy (Kazan, Russian Federation). Informed consent from blood donors was obtained in all cases. All procedures were performed in accordance with the approved guidelines. Venous blood was collected into 3.2% trisodium citrate tubes (9:1) and immediately centrifuged at room temperature at 200×*g* for 10 min to obtain platelet-rich plasma (PRP). Platelets from PRP were isolated by gel filtration at room temperature on Sepharose 2B equilibrated with Tyrode’s buffer (4 mM HEPES, 135 mM NaCl, 2.7 mM KCl, 2.4 mM MgCl_2_, 5.6 mM D-glucose, 3.3 mM NaH_2_PO_4_, 0.35 mg/ml bovine serum albumin, and pH 7.4). Platelets were counted in a hemocytometer and used within 3 h after blood collection. Cell viability was about 93–98% based on maintenance of the Δψ_m_ as determined by flow cytometry using a Δψ_m_-sensitive fluorescent dye MitoTracker DeepRed FM.

### Incubation of platelets with various activators

A total of 200,000 isolated platelets in 150 µl Tyrode’s buffer were incubated at 37 °C for various periods of time with PF4 (final concentration 10 μg/ml), HIT-like pathogenic mouse monoclonal antibodies (KKO) (final concentration 50 μg/ml), HIT-like non-pathogenic mouse monoclonal antibodies (RTO) (final concentration 50 μg/ml), and KKO/PF4 or RTO/PF4 immune complexes preformed by mixing 50 µg/ml KKO or RTO and 10 µg/ml PF4 (final concentrations). The applied concentrations of PF4 and pathogenic antibodies have been earlier shown to affect platelet functionality^49–51^. Untreated platelets were used as a negative control and platelets incubated with 10 µM calcium ionophore A23187 were used as a positive control. 4–18 independently isolated platelet preparations were studied under each experimental condition. Each experiment was performed at least in triplicate.

### Flow cytometry of platelets and platelet-derived microvesicles

In flow cytometry experiments, platelets and platelet-derived microvesicles were identified by labeling them with PE-conjugated monoclonal antibodies to CD41 (platelet integrin’s subunit αIIb). After incubation under various experimental conditions, platelets (150 µl) were mixed with 300 µl of a Ca^2+^-containing buffer (10 mM HEPES, 140 mM NaCl, 2.5 mM CaCl_2_, and pH 7.4) to ensure the binding of Annexin V-FITC that was used as a marker of phosphatidylserine expressed on the surface of platelets and microvesicles. To measure the ΔΨ_m_ platelets were labeled with a ΔΨ_m_-sensitive fluorescent dye MitoTracker DeepRed FM. To assess platelet activation, the surface expression of P-selectin was measured using PE-conjugated anti-human CD62P antibodies. To evaluate the activity of caspases 3 and 7, platelets were incubated with CellEventTM Caspase-3/7 Green Detection Reagent (500 nM final concentration) for 30 min following incubation and 1 μM SYTOX® AADvanced™ dead cell stain for 5 min before the end of the incubation (total time of incubation 90 min) under various experimental conditions and analyzed using flow cytometry.

Platelets were gated by their FSC/SSC characteristics after size-based calibration with 1 μm, 2 μm, and 4 μm polystyrene beads and by their binding of anti-CD41-PE-labeled antibodies (Figs. S1A, S2). Platelet-derived microvesicles were identified and quantified as the events that reflected a platelet-specific marker CD41 (platelet integrin’s subunit αIIb) and were characterized by forward light scatter (FSC) smaller than 1 µm (Fig. S2). Unlabeled platelets and microvesicles were used as controls to their gating in the corresponding dot plots. For each sample analyzed, 30,000 events were collected using a FacsCalibur flow cytometer (BD Biosciences, USA) equipped with an argon laser (*λ* = 488 nm) and a diode red laser (*λ* = 635 nm). The data were analyzed using CellQuest Pro (BD Biosciences) and FlowJo software.

### Measurement of ATP content in platelets

Isolated platelets (10^7^/ml) in Tyrode’s buffer were incubated for various time intervals with the immune complex KKO/PF4 in the presence or absence of 2 mM CaCl_2_. After the incubation, platelets were spun down for 5 min at 2000g and lysed with 0.2% Triton X-100 in Tyrode’s buffer for 20 min at room temperature with continuous shaking. Debris was removed from the lysates by centrifugation at 8000 *g* for 10 min. ATP concentration in the platelet lysates was measured by plate reader Infinite 200 PRO (Tecan, Switzerland) using an ATP Bioluminescent Assay Kit according to the manufacturer’s instructions (Sigma-Aldrich, USA). The ATP concentration was normalized by the protein content in the platelet lysates determined with a BCA reagent.

### Calpain activity

Gel-filtered human platelets (200,000 in 150 μl Tyrode’s buffer containing 3 mM CaCl_2_) were preincubated with 100 µM Calpain Substrate IV at 37 °C for 30 min followed by incubation for 15, 60, and 180 min at 37° with the preformed immune complexes KKO/PF4 and RTO/PF4 as well as PF4, KKO, and RTO alone at the concentrations shown above. The EDANS fluorescence intensity was measured at 380 ± 20 nm excitation and 465 ± 30 nm emission wavelengths on a multimode plate reader Infinite F Plex (Tecan, Switzerland). Platelets incubated with 100 µM calcium ionophore A23187 were used as a positive control, and untreated platelets were used as a negative control.

### Scanning electron microscopy

After incubation under various experimental conditions, platelets were fixed by 2% glutaraldehyde solution in 0.05 M cacodylate buffer containing 0.15 M NaCl (pH 7.4) for 60 min at room temperature. Fixed platelets were layered on a carbon filter with 0.4 or 0.1 µm pore size (MilliporeSigma, USA) and centrifuged at 150 g for 15 min. Samples were washed three times with the cacodylate buffer and dehydrated serially in ascending concentrations of ethanol and dried overnight with hexamethyldisilazane. A ~15-nm film of gold–palladium was layered on the samples using a sputter coater (Polaron e5100, Quorum Technologies, UK or Quorum Q 150T ES, UK). Scanning electron microscopy images were taken with an Quanta FEG 250 (FEI, USA) or Merlin (Carl Zeiss, Germany) at 5–10 kV.

### Transmission electron microscopy

Immediately after incubation under various experimental conditions, the platelet suspension was fixed by 2.5% glutaraldehyde in Tyrode’s buffer for 1.5 h at room temperature and then centrifuged at 1500 g for 5 min. The precipitate was washed with Tyrode’s buffer and postfixed with 1% osmium tetroxide in Tyrode’s buffer supplemented with sucrose (25 mg/ml) for 2 h. The samples were dehydrated in ascending ethanol concentrations, acetone, propylene oxide, and embedded into Epon 812. After polymerization of the samples during three days at increasing temperatures from 37 to 60 °C, ultrathin sections were cut using an Ultramicrotome-III (LKB, Sweden) and stained with saturated aqueous uranyl acetate and lead citrate. The specimens were examined using a Jem-1200 EX electron microscope (JEOL, Japan) at an operating voltage of 80 kV.

### Western blot analysis of procaspase 3 cleavage

Isolated platelets (2*10^8^/ml) in Tyrode’s buffer were incubated for 60 min under various experimental conditions in the presence or absence of 2 mM CaCl_2_. Then they were supplemented with a lysis RIPA buffer (pH 7.4) containing a proteinase and phosphatase inhibitor cocktail, and the mixture was incubated at 4 °С overnight with continuous shaking. The samples were centrifuged at 8000 × g for 10 min at 4 °С and the supernatants were collected. Cell lysates were suspended in a sodium dodecyl sulfate (SDS) sample-loading buffer, boiled for 3 min and subjected to gradient 12–20% SDS-PAGE. After protein transfer the nitrocellulose membranes with a 0.2 µm pore size (BioRad, USA) were incubated overnight at 4 °C with antibodies against human (pro)caspase 3 (Millipore Sigma, USA, cat. #MAB4703, clone 4–1–18). To detect the primary antibodies, blots were incubated with the secondary goat horseradish peroxidase-conjugated anti-mouse IgG antibodies (Invitrogen, USA). Blots were analyzed using a ChemiDoc Xrs + System (BioRad, USA).

### Statistical analysis

Each measurement was performed not less than in three repeats on platelets isolated from different donors. Statistical analyses were performed using Microsoft Excel and Prism 5.0 software packages (GraphPad Software, San Diego). The results are expressed as mean ± standard error of mean unless otherwise indicated. After assessing normality with the Kolmogorov–Smirnov test, a two-tailed *t* test was used at a 95% confidence level, and a nonparametric Mann–Whitney test was applied for data with a non-Gaussian distribution. Categorical values such as a fraction of active platelets on electron micrographs were analyzed using a chi-square test.

## Supplementary information

Supplemental Information
