# Supplementary material for: Platelet factor 4-containing immune complexes induce platelet activation followed by calpain-dependent platelet death
Source: Cell Death Discov. 2019 Jun 24;5:106. doi: 10.1038/s41420-019-0188-0 (PMC6591288; doi:10.1038/s41420-019-0188-0)
Supplement: Supplementary file 1 — Supplemental Information [file 41420_2019_188_MOESM1_ESM.docx]

**SUPPLEMENTARY INFORMATION**

**Platelet factor 4-containing immune complexes induce platelet activation followed by calpain-dependent platelet death**

*Running title:* Heparin-induced calpain-dependent platelet death

Tatiana A. Nevzorova^a^, Elmira R. Mordakhanova^a^, Amina G. Daminova^a,b^, Anastasia A. Ponomareva^a,b^, Izabella A. Andrianova^a^, Giang Le Minh^a^, Lubica Rauova^c,d^, Rustem I. Litvinov^a,e^ and John W. Weisel^e,*^

^a^*Institute of Fundamental Medicine and Biology, Kazan Federal University, 18 Kremlyovskaya St., Kazan 420008, Russian Federation*

^b^*Kazan Institute of Biochemistry and Biophysics, FRC Kazan Scientific Center of RAS, 2/31 Lobachevsky str., Kazan 420111, Russian Federation*

^c^*Children’s Hospital of Philadelphia, 3401 Civic Center Blvd, Philadelphia, PA 19104, USA*

^d^*Department of Pediatrics, University of Pennsylvania Perelman School of Medicine, 3401 Civic Center Blvd, Philadelphia, PA 19104, USA*

^e^*Department of Cell and Developmental Biology, University of Pennsylvania Perelman School of Medicine, 421 Curie Boulevard, Philadelphia, PA 19104, USA*

**Table S1.** Diameter of platelet bodies (in micrometers) based on scanning electron microscopy

| *Experimental conditions* | *Time of incubation* | |
| --- | --- | --- |
|  | *15 min* | *60 min* |
| Untreated platelets | 2.34±0.07 | 2.42±0.03 |
| KKO/PF4 | 1.64±0.02*** | 1.71±0.03*** |
| KKO | 1.83±0.03*** | 1.75±0.03*** |
| PF4 | 2.09±0.06* | 2.14±0.03*** |
| A23187 | 1.78±0.05*** | 1.87±0.04*** |

Number of individual platelets analyzed varied from 100 to 250 at each experimental condition.

*p<0.05, ***p<0.001 compared to control untreated platelets.


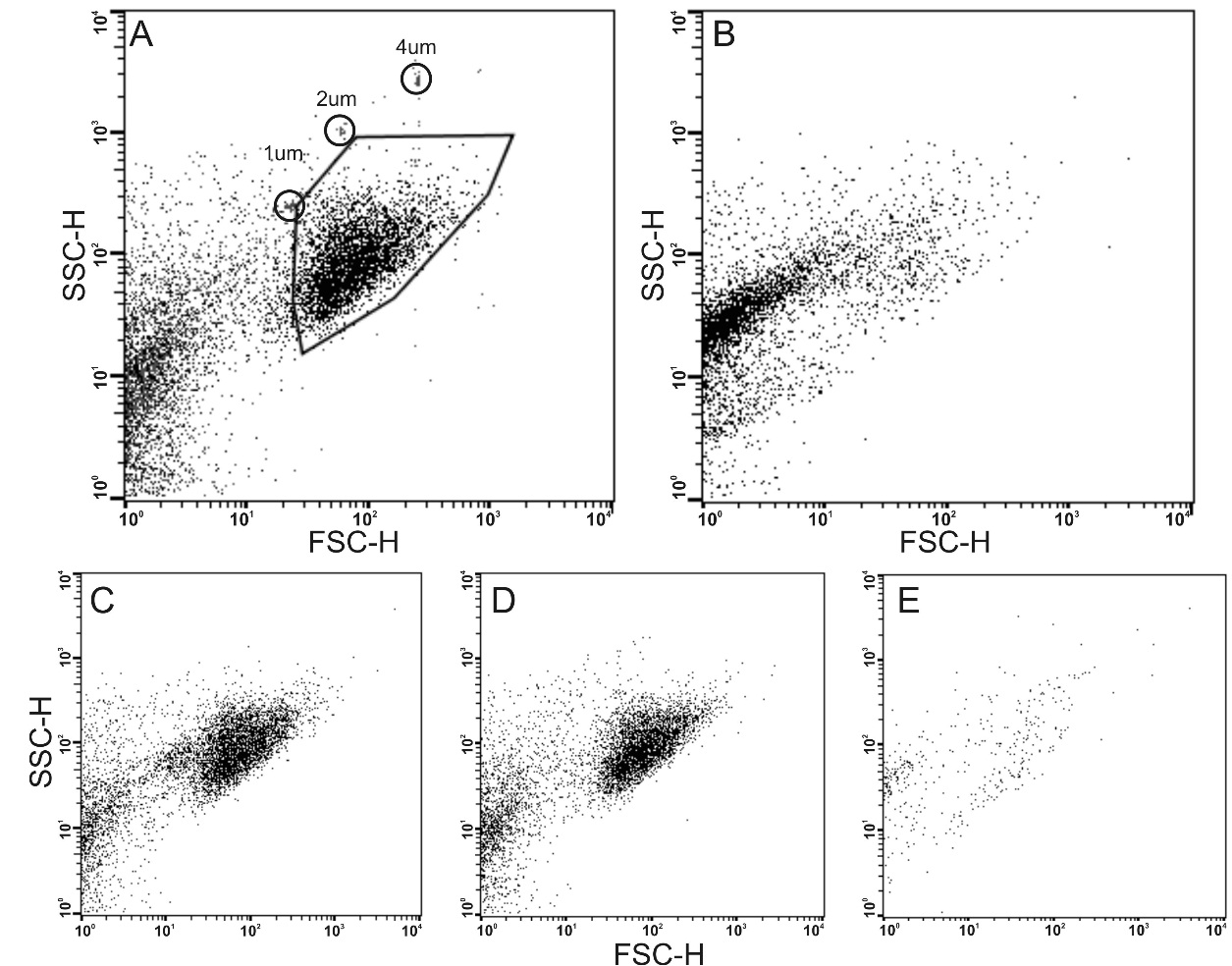


**Figure S1.** **Flow cytometry of isolated platelets** in a two-channel FSC vs. SSC mode (distribution by size and granularity, respectively). Platelets were incubated at 37°C for 15 min under various experimental conditions: (**A**) untreated platelets (negative control) showing the corresponding gate; (**B**) treated with KKO/PF4; (**C**) treated with KKO; (**D**) treated with PF4; (**E**) treated with Ca^2+^-ionophore A23187. Final concentrations: 10 µg/ml PF4, 50 µg/ml KKO and RTP, 10 µM Ca^2+^-ionophore. Treatment with KKO/PF4 and A23187 resulted in a substantial reduction of the gated platelet populations.


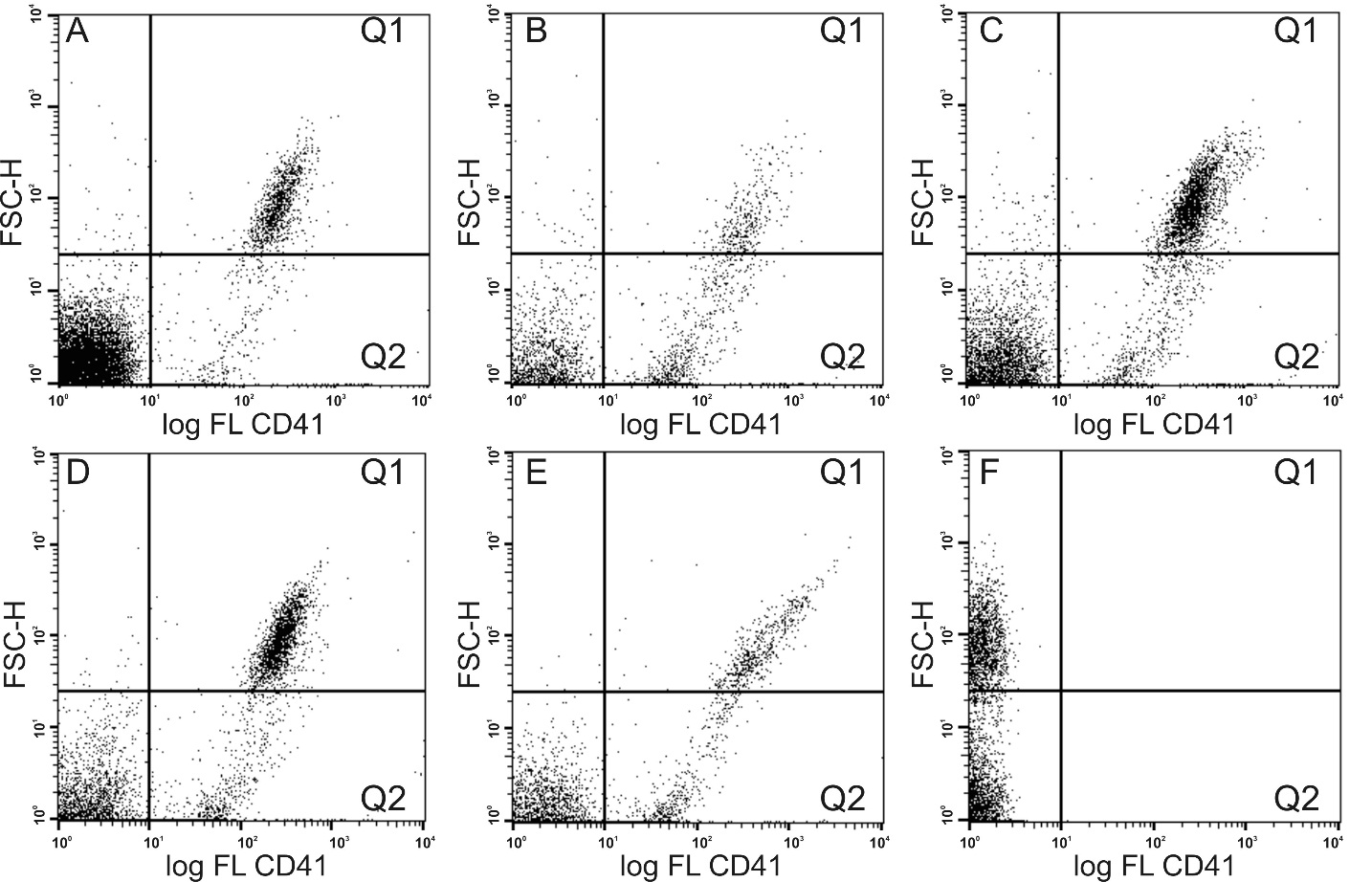


**Figure S2.** **Representative flow cytometry dot plots** showing CD41-positive platelets (Q1 quadrant) and CD41-positive platelet-derived microvesicles (Q2 quadrant) after incubation of isolated platelets under various experimental conditions. Platelets were labeled with PE-conjugated antibodies to human CD41 (log FL CD41 vs. FSC): (**A**) untreated platelets (negative control); (**B**) treated with KKO/PF4; (**C**) KKO; (**D**) PF4; (**E**) Ca^2+^-ionophore A23187; and (**F**) control untreated platelets without labeling.

**
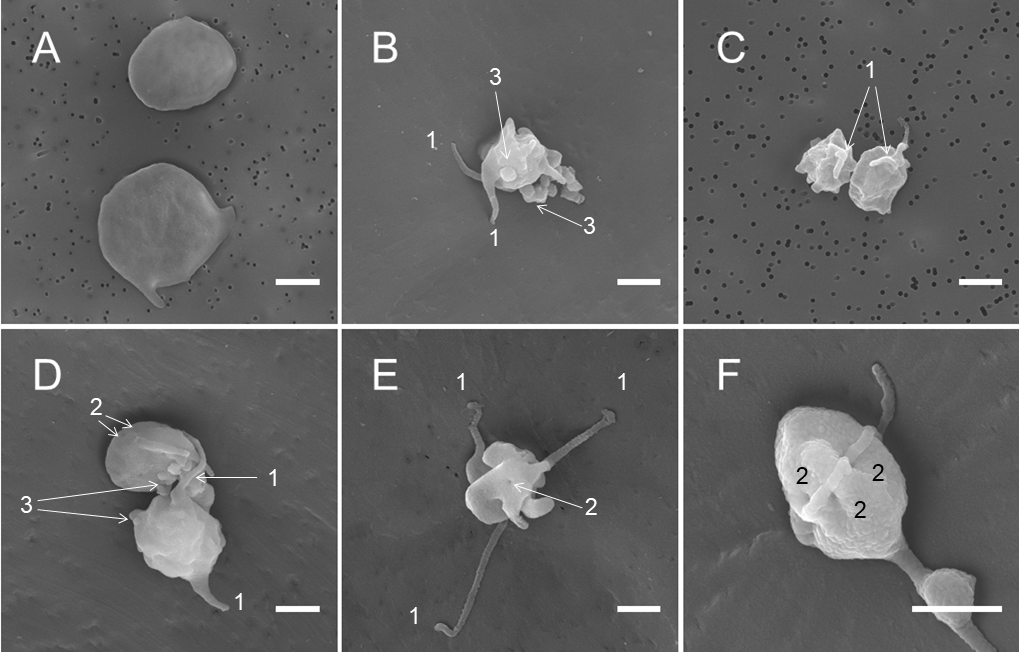
**

**Figure S3. Representative scanning electron micrographs of platelets incubated for 60 min at 37°C under various experimental conditions:** (**A**) control untreated resting platelets; (**B**) a platelet treated with KKO/PF4; (**C**) platelets treated with KKO alone; (**D**) platelets treated with PF4 alone, and (**E, F**) a platelet treated with Ca^2+^-ionophore A23187. Final concentrations: 10 µg/ml PF4, 50 µg/ml KKO, 12 µM Ca^2+^-ionophore. Arrows and numbers indicate: 1 – filopodia/pseudopodia, 2 – pores of the open canalicular system, 3 – blebs and knobs. Magnification bars: 1 µm.

**
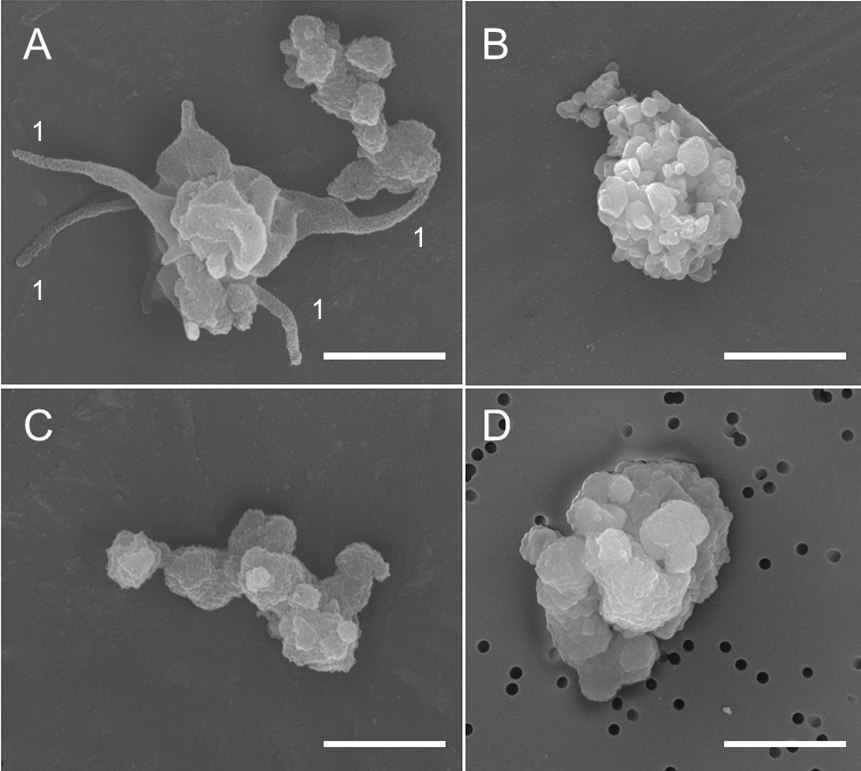
**

**Figure S4. Representative scanning electron micrographs of aggregated microvesicles in platelet preparations treated with KKO/PF4 for 60 min at 37°C.** (**A**) An activated platelet and an aggregate of microvesicles; (**B**) a multivesicular particle; (**C, D**) aggregated microvesicles. Number 1 indicates filopodia. Magnification bars: 1 µm.


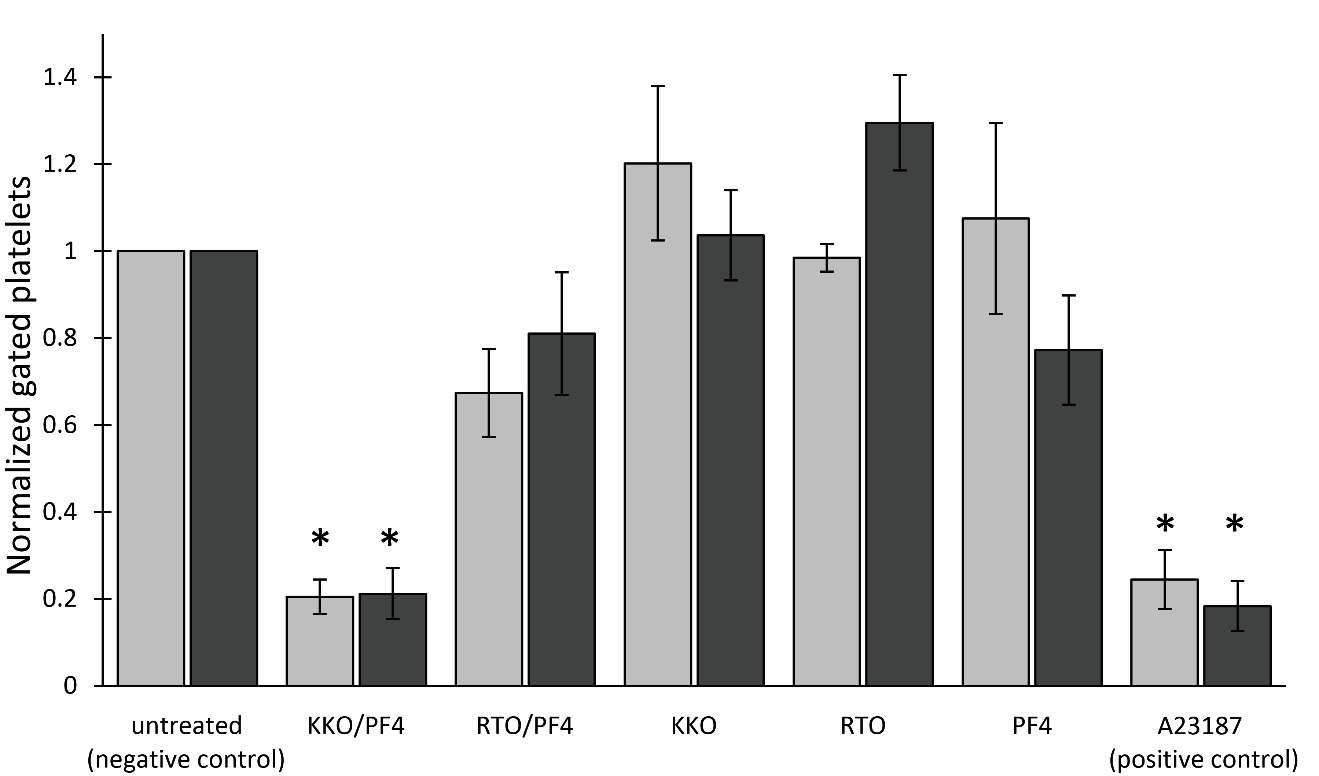


**Figure S5. Relative number of the size-gated platelets normalized by the number of platelets in the negative control.** Platelets were incubated for 15 min (light bars) and 60 min (dark bars) under the following conditions: untreated platelets (n=15/13 for 15 min/60 min, respectively), treated with KKO/PF4 (n=15/13), RTO/PF4 (n=3/3), KKO (n=15/13), RTO (n=3/3), PF4 (n=15/13), and Ca^2+^-ionophore A23187 (n=13/12). Final concentrations: 10 µg/ml PF4, 50 µg/ml KKO and RTP, 10 µM Ca^2+^-ionophore. *n* is the number of experiments with platelets isolated from independent donors. *P<0.05 compared to a corresponding negative control. The differences between 15 min and 60 min are insignificant.


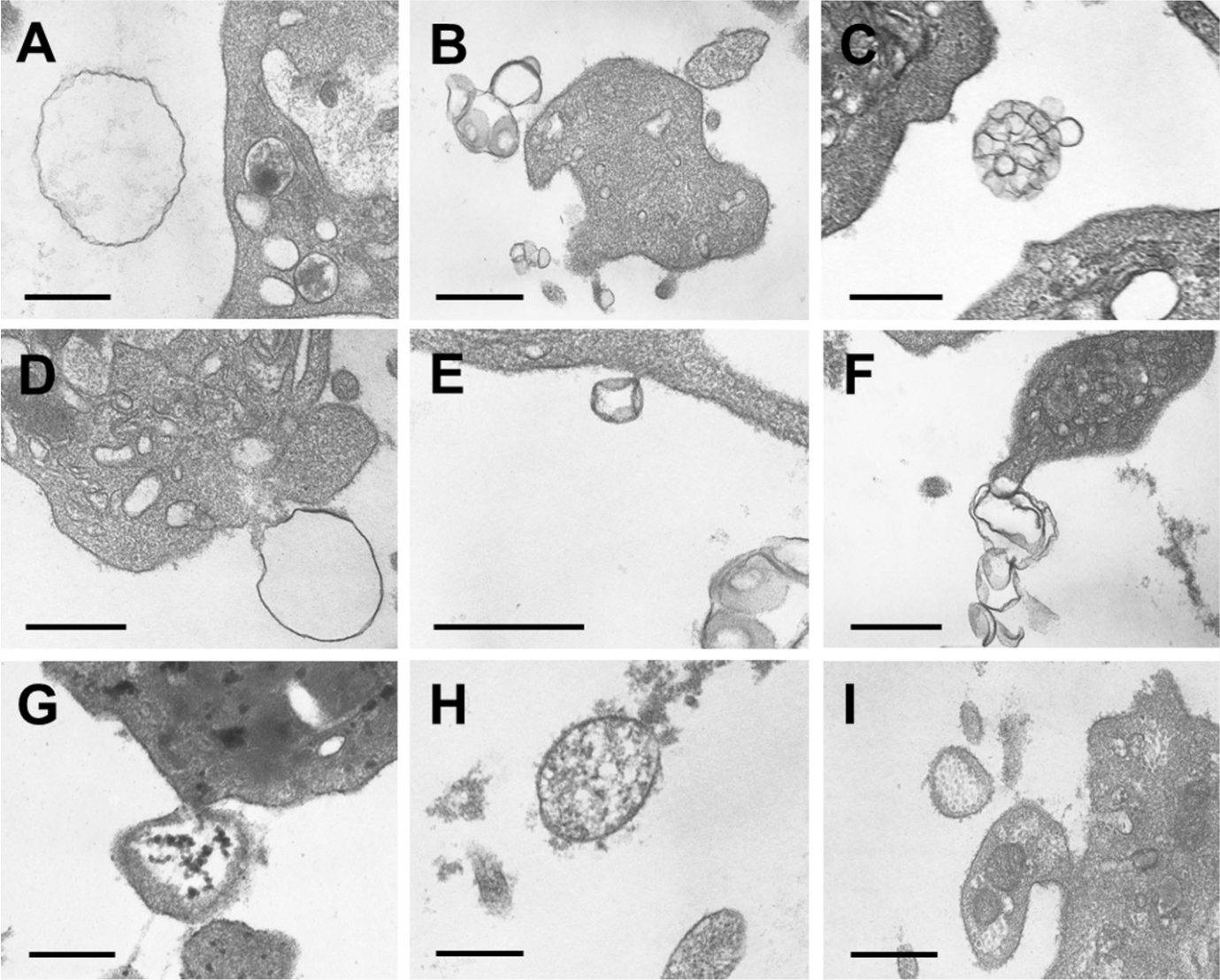


**Figure S6. Structural diversity of platelet-derived microvesicles** formed after incubation of platelets with the immune complex KKO/PF4 for 60 min at 37°C. Magnification bars: 0.5 μm.


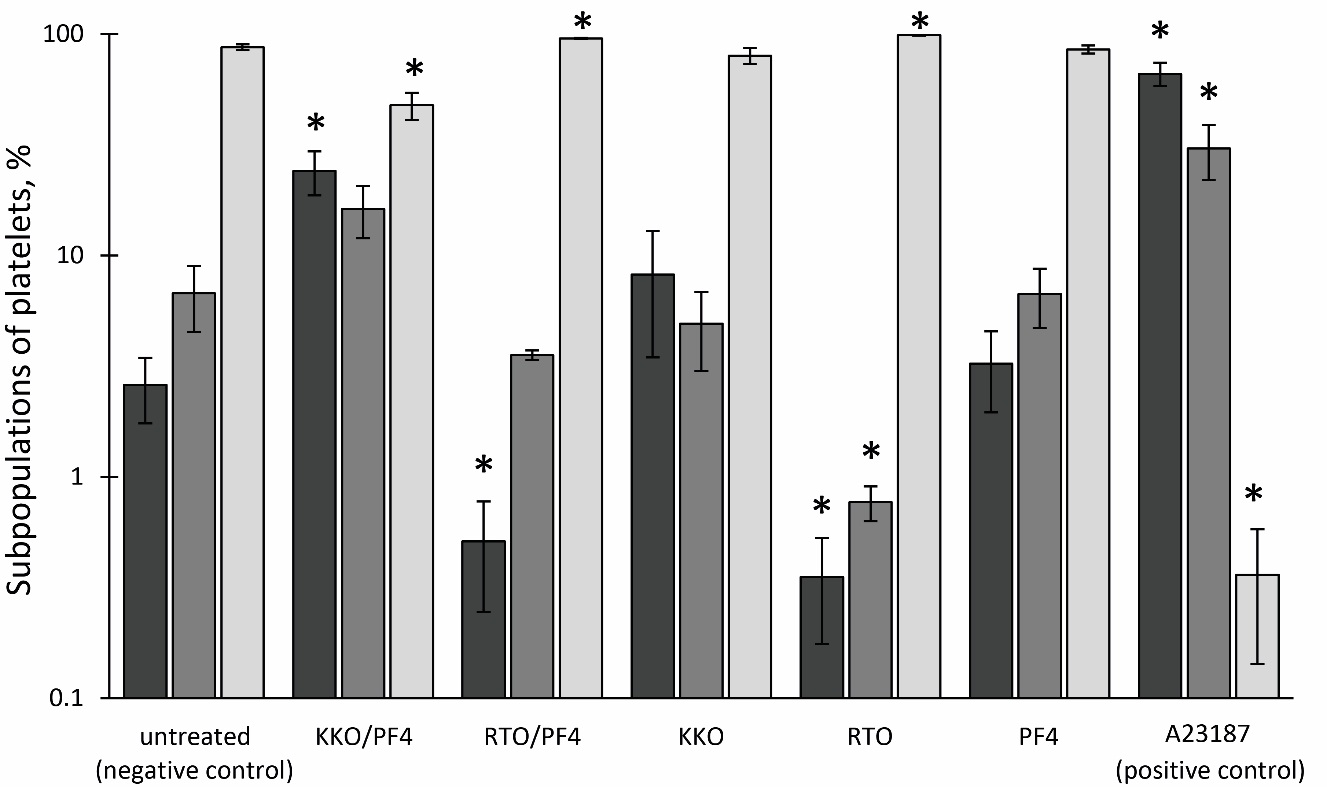


**Figure S7. Subpopulations of platelets double-stained with a ΔΨ_m_-sensitive dye MitoTrackerDeepRed and FITC-Annexin V normalized by the total number of gated platelets taken as 100%.** Platelets were segregated into 3 groups: MitoTracker-negative/Annexin V-positive (dark bars), MitoTracker-positive/Annexin V-positive (grey bars), and MitoTracker-positive/Annexin V-negative (light bars). Platelets were incubated 60 min under the following conditions: untreated platelets (n=13); treated with KKO/PF4 (n=13), RTO/PF4 (n=3), KKO (n=13), RTO (n=3), PF4 (n=13), and Ca^2+^-ionophore A23187 (n=12). Final concentrations: 10 µg/ml PF4, 50 µg/ml KKO and RTP, 10 µM Ca^2+^-ionophore. n is the number of experiments with platelets isolated from independent donors. *P<0.05 compared to a corresponding negative control. The differences between 15 min and 60 min are insignificant.


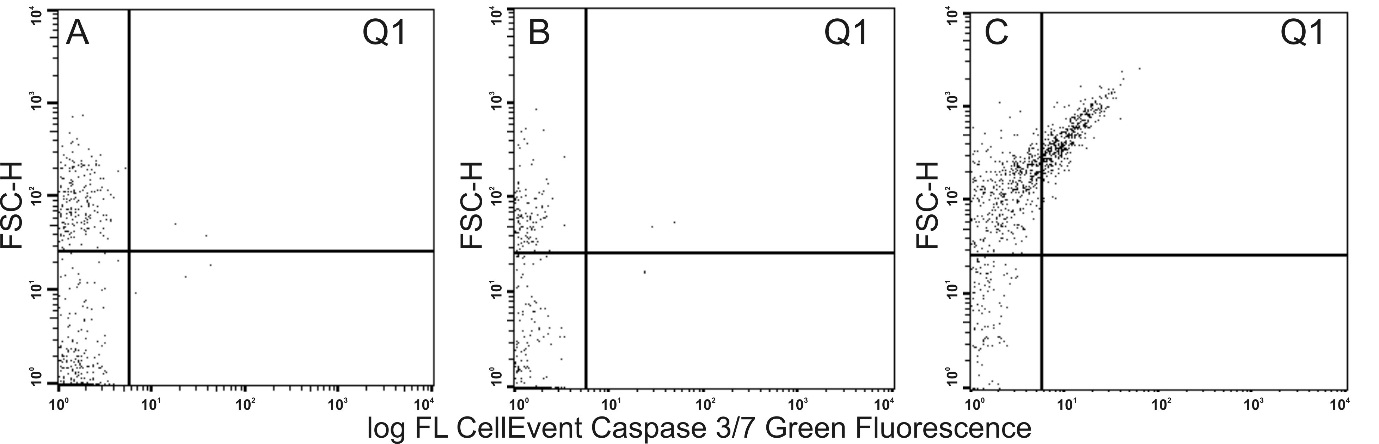


**Figure S8.** **Representative flow cytometry dot plots** showing the absence or presence of caspase 3/7-positive platelets (Q1 quadrant) under various experimental conditions. To reveal the caspase activity, platelets were incubated with CellEventTM caspase-3/7 Green Detection Reagent. (**A**) Untreated platelets (negative control); (**B**) treated with KKO/PF4 (50 µg/ml + 10 µg/ml); (**C**) treated with Ca^2+^-ionophore A23187 (10 μM). No detectable caspase activity in A and B, while in C the caspase activity is visualized in Q1. The same negative results as shown in A and B were obtained with platelets treated with PF4/RTO as well as with PF4 and KKO alone (not shown).

**
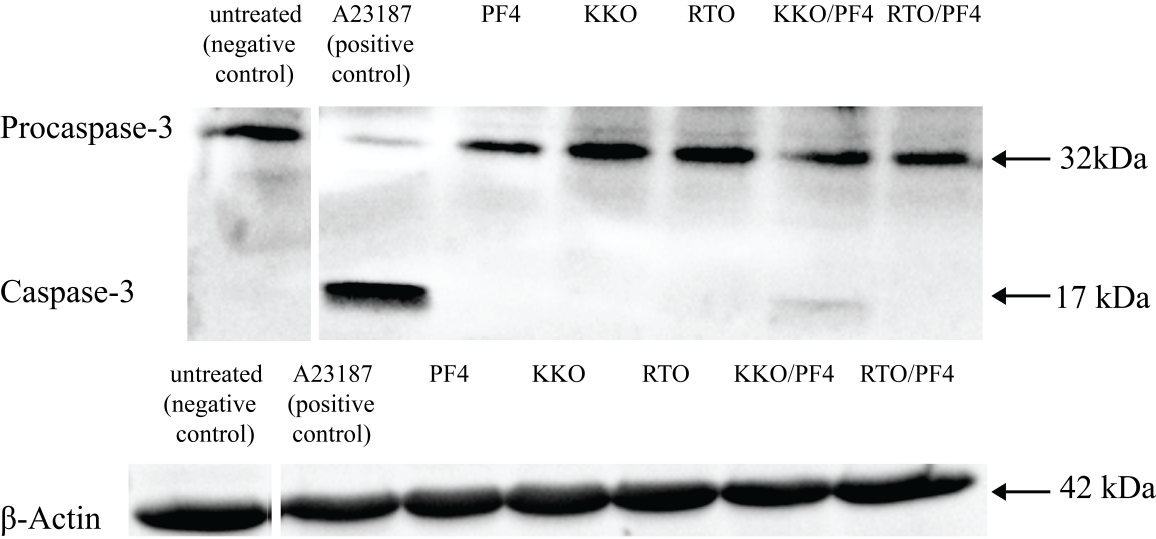
**

**Figure S9. Western blot analysis of procaspase 3 cleavage.** Platelet lysates were obtained from control untreated platelets (lane 1) and platelets treated with Ca^2+^-ionophore A23187 (lane 2), PF4 (lane 3), KKO (lane 4), (RTO lane 5), KKO/PF4 (lane 6) and RTO/PF4 (lane 7). A faint band of cleaved caspase 3 (17 kDa) is present in platelets treated with the KKO/PF4 complexes. Calcium ionophore A23187 used as a positive control induced almost complete conversion of the procaspase 3 (32 kDa) to the active cleaved caspase 3 (17 kDa). A representative Western blot is shown from 3 independent experiments with reproducible results.
